# Supplementary material for: Gender differences in the perception of leptospirosis severity, behaviours, and Leptospira exposure risk in urban Brazil: a cross-sectional study
Source: medRxiv. 2024 Apr 30:2024.04.28.24306445. Preprint. [Version 1] doi: 10.1101/2024.04.28.24306445 (PMC11092738; doi:10.1101/2024.04.28.24306445)
Supplement: Supplement 1 [file NIHPP2024.04.28.24306445v1-supplement-1.pdf]

Supplementary Information

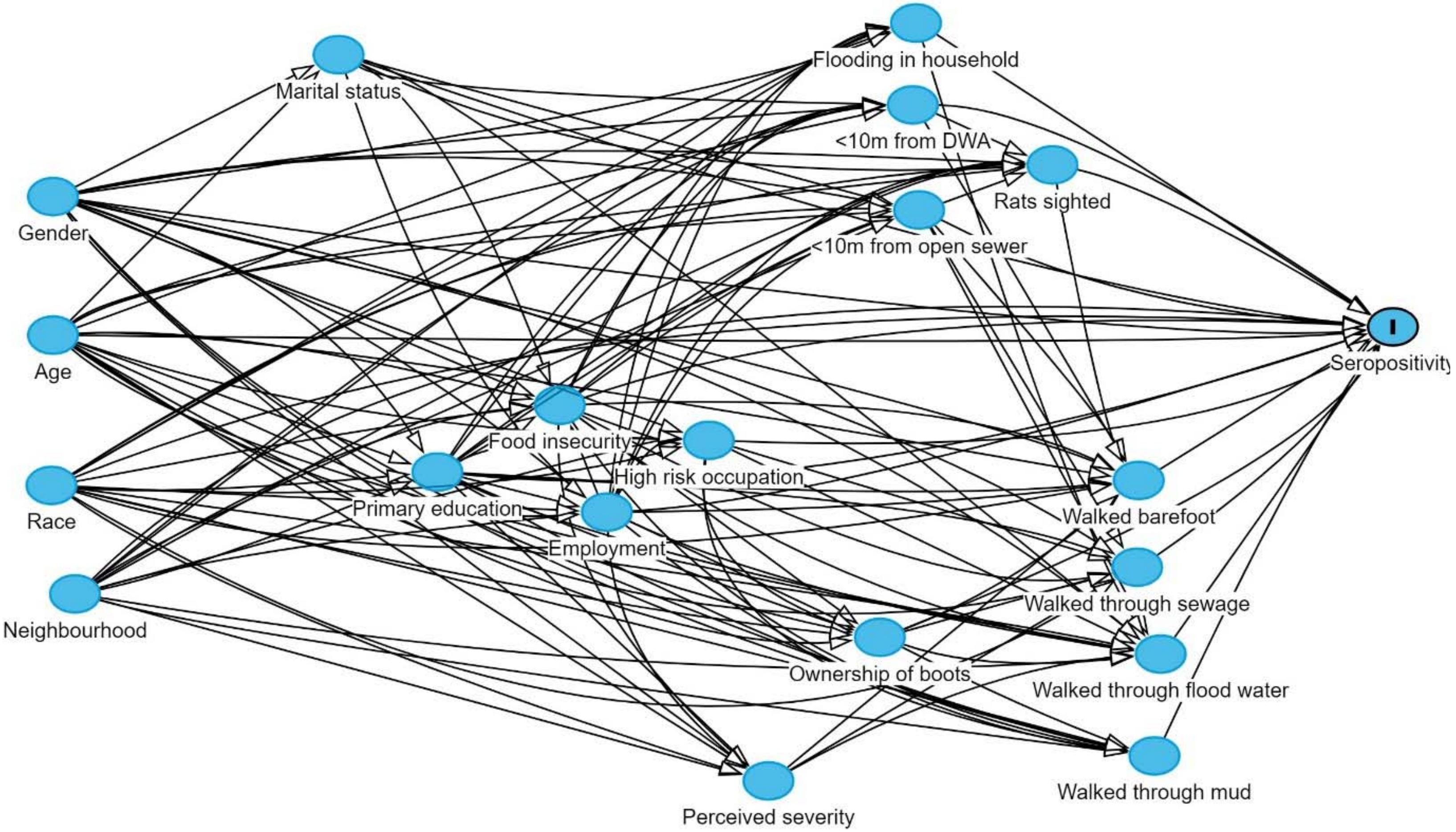

Supplementary Information 1: Full version of the DAG used in analysis, also available at https://dagitty.net/dags.html?id=XxPTXytr#. DWA: Domestic waste accumulation

Supplementary Information 2a: Sex-disaggregated descriptive analysis of seroprevalence across risk-factors.

	Females		Males	
	Total	Seroprevalence n (%)	Total	Seroprevalence n (%)
**Sociodemographic**				
Age group (years)	481		280	
18-30		8 (6.5)		5 (6.4)
31-45		15 (8.9)		13 (12.9)
46-60		12 (10.0)		13 (18.3)
>60		10 (14.7)		10 (33.3)
Race	481		280	
Black		22 (9.1)		23 (15.9)
Other		4 (12.1)		1 (6.7)
Pardo		19 (9.2)		17 (14.2)
Completion of primary education	481		280	
Yes		6 (5.9)		6 (10.2)
No		39 (10.3)		35 (15.8)
Employment status (as of previous week)	480		279	
Unemployed		27 (9.8)		19 (20.2)
Formal		6 (9.2)		12 (12.8)
Informal		12 (8.6)		10 (11.0)
Occupation	480		279	
High-risk		4 (8.2)		10 (21.7)
Other		14 (9.0)		12 (8.6)
Unemployed		27 (9.8)		19 (20.2)
**Perceptions of leptospirosis**				
Perceived severity of leptospirosis	474		272	
Less serious		9 (14.3)		9 (26.5)
Extremely serious		36 (8.8)		31 (13.0)
**Behaviours (in last 6 months)**				
Walked through flood water	474		272	
Rarely		35 (10.1)		32 (15.1)
Frequently		10 (7.9)		9 (15.0)
Walked through sewage water	474		273	
Rarely		38 (10.2)		31 (14.6)
Frequently		7 (7.0)		10 (16.4)
Could wear boots during flooding	475		273	
No		37 (9.5)		21 (15.3)
Yes		8 (9.3)		20 (14.7)
Walked barefoot	474		273	
Rarely		33 (9.0)		29 (14.0)
Frequently		12 (11.1)		12 (18.2)
Walked through mud	475		273	
Rarely		32 (9.0)		29 (14.8)
Frequently		13 (10.8)		12 (15.6)

Supplementary Information 2b: Sex-disaggregated univariable logistic regression analysis.

				**Sex-disaggregated**					
	**Combined**			Female-restricted			Male-restricted		
**Exposure**	n	OR (95% CI)	*p*-value	n	OR (95% CI)	*p*-value	n	OR (95% CI)	*p*-value
**Sociodemographics**									
Gender	761								
Female		REF	REF		N/A			N/A	
Male		1.66 (1.06, 2.61)	0.027						
Age (increase per year)	761	1.02 (1.01, 1.04)	0.001	481	1.02 (1.00, 1.05)	0.12	280	1.04 (1.01, 1.06)	0.002
Race	761			481			280		
Black		REF	REF		REF	REF		REF	REF
Other		0.88 (0.33, 2.34)			1.45 (0.34, 6.14)	0.6		0.38 (0.05, 3.02)	0.4
Pardo		0.94 (0.59, 1.49)	0.8		1.00 (0.45, 2.22)	>0.9		0.88 (0.44, 1.73)	0.7
Primary education	761			481			280		
Completed		REF	REF		REF	REF		REF	REF
Incomplete		1.75 (0.92, 3.30)	0.086		2.01 (0.68, 5.96)	0.2		1.66 (0.66, 4.16)	0.3
Employment status (in the last week)	759			480			279		
Formally employed		REF	REF		REF	REF		REF	REF
Unemployed		1.11 (0.62, 1.99)	0.7		1.06 (0.33, 3.39)	>0.9		1.73 (0.79, 3.81)	0.2
Informally employed		0.83 (0.43, 1.60)	0.6		0.86 (0.23, 3.15)	0.8		0.84 (0.35, 2.06)	0.7
Occupation (among employed)	389			204			185		
Other		REF	REF		REF	REF		REF	REF
High-risk		1.78 (0.89, 3.57)	0.1		0.90 (0.24, 2.64)	0.9		3.50 (0.41, 29.6)	0.3
**Perceptions of leptospirosis**									
Perceived severity of leptospirosis	746			474			272		
Less serious		REF	REF		REF	REF		REF	REF
Extremely serious		0.51 (0.29, 0.89)	0.019		0.54 (0.20, 1.47)	0.2		0.42 (0.18, 0.97)	0.043
**Behaviours in the last 6 months**									
Walked through floodwater	746			474			272		
Rarely or never		REF	REF		REF	REF		REF	REF
Frequently		0.84 (0.49, 1.43)	0.5		0.71 (0.26, 1.89)	0.5		0.99 (0.45, 2.21)	>0.9

Walked through sewage water	747			474			273		
Rarely or never		REF	REF		REF	REF		REF	REF
Frequently		0.88 (0.50, 1.55)	0.7		0.55 (0.17, 1.78)	0.3		1.14 (0.53, 2.49)	0.7
Could wear boots during flooding	748			475			273		
Yes		REF	REF		REF	REF		REF	REF
No		0.86 (0.53, 1.39)	0.5		1.02 (0.36, 2.90)	>0.9		1.05 (0.54, 2.04)	0.9
Walked barefoot outside of home	747			474			273		
Rarely or never		REF	REF		REF	REF		REF	REF
Frequently		1.32 (0.80, 2.19)	0.3		1.41 (0.54, 3.68)	0.5		1.36 (0.65, 2.85)	0.4
Walked through mud	748			475			273		
Rarely or never		REF	REF		REF	REF		REF	REF
Frequently		1.17 (0.71, 1.92)	0.5		1.31 (0.53, 3.27)	0.6		1.06 (0.51, 2.21)	0.9

Supplementary Information 2c: GAMs of age with seroprevalence response variable. GAMs were built using univariable models, and shaded area corresponds to 95%CI.

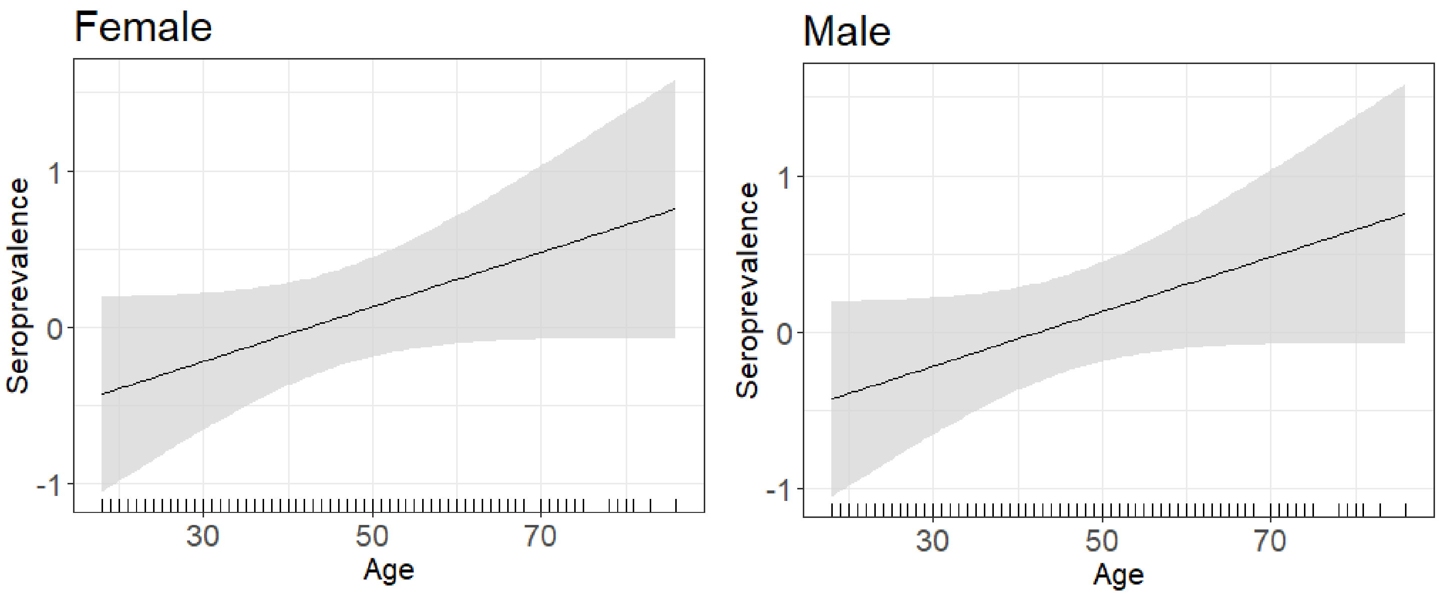

Supplementary Information 3a: Descriptive analysis of prevalence of behaviours across perceived severity.

	Female n, (%)			Male n, (%)		
**Behaviour (in last 6 months)**	**n**	**Less serious n=63**	**Extremely serious n = 411**	**n**	**Less serious n=38**	**Extremely serious n = 238**
Walked through flood water	474			271		
Rarely		45 (71.4)	303 (73.7)		28 (82.4)	184 (77.6)
Frequently		18 (28.6)	108 (26.3)		6 (17.6)	53 (22.4)
Walked through sewage water	474			272		
Rarely		48 (76.2)	326 (79.3)		24 (70.6)	188 (79.0)
Frequently		15 (23.8)	85 (20.7)		10 (29.4)	50 (21.0)
Could wear boots during flooding	474			272		
No		11 (17.5)	75 (18.2)		15 (44.1)	121 (50.8)
Yes		52 (82.5)	336 (81.8)		19 (55.9)	117 (49.2)
Walked barefoot	474			272		
Rarely		50 (79.4)	316 (76.9)		20 (58.8)	187 (78.6)
Frequently		13 (20.6)	95 (23.1)		14 (41.2)	51 (21.4)
Walked through mud	474			272		
Rarely		44 (69.8)	310 (75.4)		26 (76.5)	170 (71.4)
Frequently		19 (30.2)	101 (24.6)		8 (23.5)	68 (28.6)

Supplementary Information 3b: Sex-disaggregated univariable logistic regression analysis of the association of perceived severity with high-risk behaviours

**Perceived severity of leptospirosis**	**Behaviour** Walked through flood water			Walked through sewage water			Could wear boots during flooding			Walked barefoot			Walked through mud		
	n	OR (95% CI)	*p*	n	OR (95% CI)	*p*	n	OR (95% CI)	*p*	n	OR (95% CI)	*p*	n	OR (95% CI)	*p*
**Combined**	745			272			746			746			272		
Less serious		REF			REF			REF			REF			REF	
Extremely serious		1.01 (0.53, 1.86)	>0.9		0.70 (0.39, 1.24)	0.2		0.85 (0.49, 1.48)	0.6		0.73 (0.42, 1.25)	0.2		0.73 (0.25, 2.11)	0.6
**Sex-disaggregated**															
Female-restricted	474			474			474			474			474		
Less serious		REF			REF			REF			REF			REF	
Extremely serious		0.83 (0.36, 1.93)	0.7		0.70 (0.29, 1.69)	0.4		0.95 (0.47, 1.90)	0.9		1.23 (0.57, 2.67)	0.6		0.64 (0.28, 1.48)	0.3
Male-restricted	271			272			272			272			272		
Less serious		REF			REF			REF			REF			REF	
Extremely serious		1.51 (0.38, 6.03)	0.6		0.61 (0.24, 1.52)	0.3		0.71 (0.25, 2.01)	0.5		0.39 (0.18, 0.82)	0.014		1.41 (0.47, 4.28)	0.5

Supplementary 3c: GAMs of age with high-risk behaviour response variables, and position of knots used to model non-linear relationships. GAMs were built using univariable models, and shaded area corresponds to 95%CI.

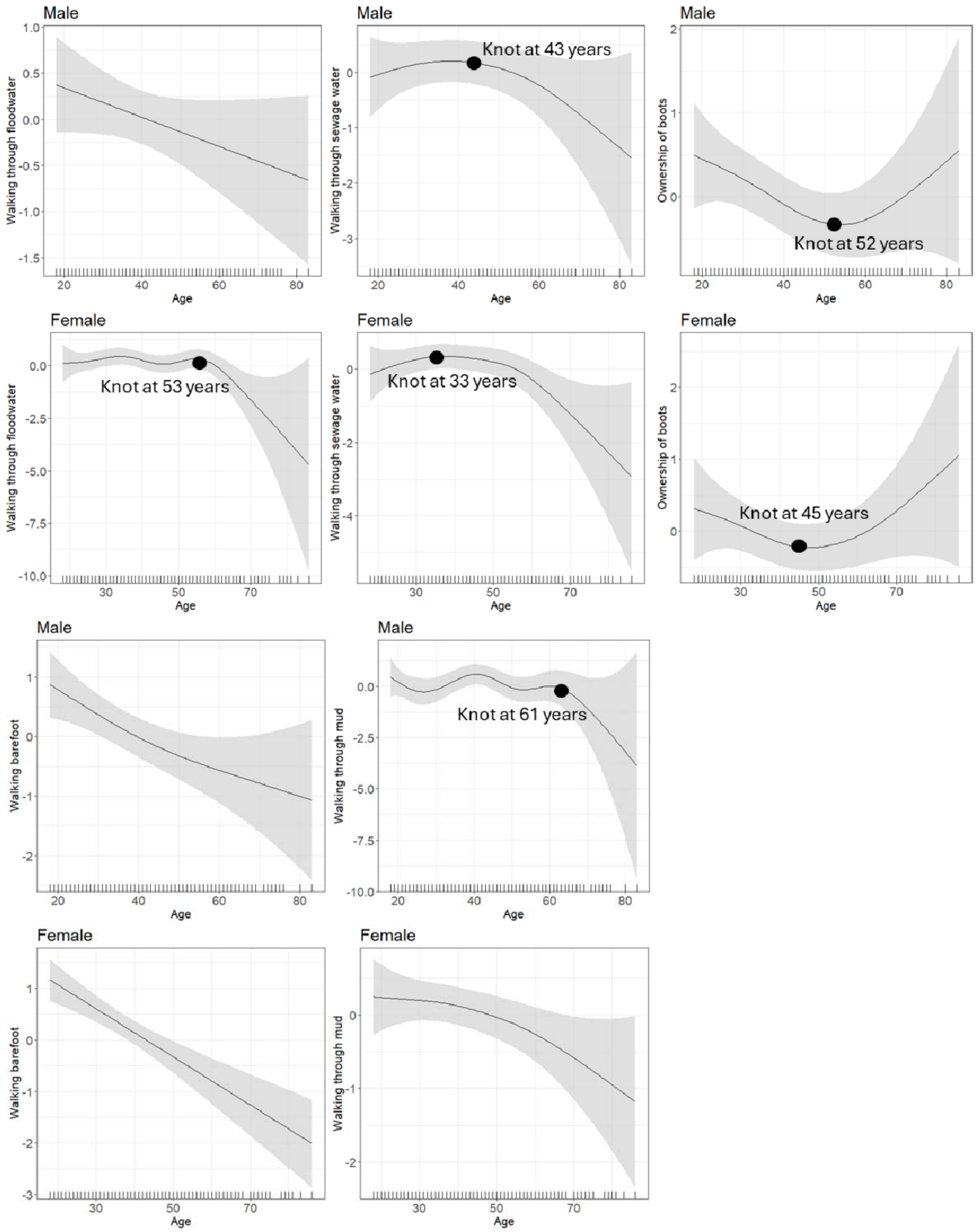

Supplementary Information 3d: Total causal effect estimates for the effect of perceived severity on the risk of performing high-risk behaviours, shown for the combined and sex-disaggregated multivariable logistic regression models.

**Perceived severity of leptospirosis**	**Behaviour** Walked through flood water			Walked through sewage water			Could wear boots during flooding			Walked barefoot outside the home			Walked through mud		
	n	aOR (95% CI)*	*p*	n	aOR (95% CI)*	*p*	n	aOR (95% CI)**	*p*	n	aOR (95% CI)***	*p*	n	aOR (95% CI)*	*p*
**Combined**	724			725			725			726			725		
Less serious		REF			REF			REF			REF			REF	
Extremely serious		0.96 (0.45, 1.67)	>0.9		0.67 (0.40, 1.15)	0.13		0.84 (0.44, 1.60)	0.6		0.64 (0.37, 1.13)	0.12		0.79 (0.45, 1.38)	0.4
**Sex-disaggregated**															
Female-restricted	462			462			462			463			462		
Less serious		REF			REF			REF			REF			REF	
Extremely serious		0.84 (0.38, 1.86)	0.7		0.80 (0.41, 1.62)	0.5		0.87 (0.42, 1.81)	0.7		1.15 (0.53, 2.50)	0.7		0.76 (0.37, 1.58)	0.5
Male-restricted	262			263			263			263			263		
Less serious		REF			REF			REF			REF			REF	
Extremely serious		1.00 (0.32, 3.11)	>0.9		0.41 (0.17, 1.00)	0.049		0.75 (0.24, 2.63)	0.7		0.24 (0.08, 0.76)	0.015		0.83 (0.26, 2.66)	0.6
Test for interaction				0.7		0.3			0.4			0.007			0.4

Adjustments: *neighbourhood, age, gender, race, food insecurity, employment, education; **age, gender, race, food insecurity, employment, education; ***neighbourhood, age, gender, race, food insecurity, employment, education. Gender was removed as a confounder in the sex-disaggregated models.
